# Seven Years Study of the Seasonal Dynamics of Zooplankton Communities in a Large Subtropical Floodplain Ecosystem: A Test of the PEG Model

**DOI:** 10.3390/ijerph19020956

**Published:** 2022-01-15

**Authors:** Baogui Liu, Jiayi Wu, Yang Hu, Guoxiang Wang, Yuwei Chen

**Affiliations:** 1 School of Geography, Nanjing Normal University, Nanjing 210023, China; bgliu@njnu.edu.cn; 2 School of Environmental, Nanjing Normal University, Nanjing 210023, China; wjy_jiayi@126.com (J.W.); 26200320@njnu.edu.cn (Y.H.); 3 Nanchang Institute of Technology, Nanchang 330099, China

**Keywords:** Lake Poyang, water level, allochthonous sources, predation pressure, flooded hygrophytes, advection migration

## Abstract

Irregular hydrological events, according to a classic plankton ecology group (PEG) study, can generate major deviations from the standard PEG model. However, little is known about the function of hydrological factors in influencing the seasonal dynamics of plankton. We used multivariate and Partial Least Squares Path Modeling to analyze the seasonal variation in crustacean zooplankton and related environmental factors from winter 2009 to winter 2016 in Lake Poyang, the largest freshwater lake in China. We found a distinct seasonal pattern in zooplankton development, which deviated, in part, from the PEG model, as we found indications of (1) a weaker degree of food limitation in winter and spring, likely due to high concentrations of allochthonous sources caused by decomposition of seasonally flooded hygrophytes, also affecting sediment dynamics; (2) a peak in crustacean zooplankton biomass in summer when the water level was high (and predation was lower), and where horizontal transport of zooplankton from the littoral zone to the pelagic was possibleand (3) a higher predation pressure in autumn, likely due to a shrinking water volume that left the fish concentrated in less water. The majority of these differences can be attributed to the direct or indirect impacts of physical factor variation.

## 1. Introduction

The pattern of seasonal succession has been a central theme in plankton ecology, but elucidating plankton successional trajectories and mechanisms in floodplain ecosystems remains a major challenge [1]. The flood pulse concept proposes that rivers and their fringing floodplains are integrated by strong interactions between hydrological and ecological processes [2]. Floodplain lakes (FPLs) in (sub)tropical regions can exhibit strong variations of abiotic factors owing to the occurrence of flood pulses that may influence the abiotic variables and seasonal patterns of plankton. Consequently, seasonal succession is often more complex in FPL plankton communities than in more isolated lakes [3,4], since it does not follow a simple annually repeated process of community assembly during which community interactions can be studied more concisely [5]. Most hydro-ecological research projects on FPLs have focused on assemblages of fish, benthic invertebrates and periphyton but, with a few exceptions, have neglected zooplankton communities. Hydroregime changes have, however, been recognized as the leading force in shaping the zooplankton assemblages of FPLs in Parana River [3,6], Amazon Atchafalaya River [7], Danube River [8,9], and Illinois River [4,10,11]. The water level in FPLs fluctuates between limnophase (high water level) and potamophase (low water level), which may affect the zooplankton community directly by the degree of dilution (e.g., by runoff from the upstream catchment or by precipitation) and drift and indirectly by affecting the food quantity and quality, predation pressure, suspended solids concentrations and other physical or chemical variables [12,13]. Decreasing water level may specifically increase predation pressure [14], and higher turbidity caused by fast increased water level may prevent the selection of some zooplankton species by visual predators in high predation scenarios [15], which otherwise prevent large-bodied species from dominating the community under high predation pressure scenarios. Under low predation pressure, however, large-bodied cladocerans are often more abundant in systems with a long retention time (lentic conditions), while small-bodied cladocerans and copepod nauplii prevail when the retention time is short [16,17,18]. Although the classic study of Brett and Kainz [19]) showed that phytoplankton, but not allochthonous terrestrial particulate organic carbon, sustains herbivorous zooplankton production (see also [20]), a growing number of studies have shown that both allochthonous and autochthonous make a significant contribution to zooplankton nutrient source in FPLs [21,22], but the pathways of allochthonous carbon through zooplankton food webs (e.g., subsidized indirectly by bacteria or other organisms) are complex [23,24]. Therefore, the quantity and quality of terrestrial autochthonous resources to zooplankton in FPLs may vary with water level and season in a complex way that so far is not well elucidated.

Zooplankton is also affected by seasonal variation in water temperature. Temperature directly affects zooplankton growth [25] but also indirectly by altering the quantity and quality of their food, competition, and predation patterns [26,27]. However, the seasonal response to temperature is less straightforward in FPLs than in closed lake basins due to the effect of water level changes and flow. In the floodplain of Po River, Italy, for example, zooplankton abundance was positively correlated with water temperature, but in the warmer months, it was inversely correlated with river flow (Rossetti et al., 2009).

The seasonal dynamics of lake plankton communities have been partially captured in a conceptual framework, the Plankton Ecology Group (hereafter PEG) model [5,28]. The PEG model, established in 1977, is a verbal model describing the patterns and driving factors of seasonal plankton succession [29]. The PEG model was first published by Sommer and Gliwicz [28]). The model and its recent update (Sommer et al., 2012) have been extensively applied in studies of lakes in different climatic regions [30,31] and describe the seasonal succession of phytoplankton and zooplankton in 24 sequential steps [28]. To what extent the PEG model can be transferred to FPL, characterized by a seasonal variation in current, retention time, and water level, is uncertain. Usually, the seasonal succession of plankton in FPLs is regarded as unpredictable as it may be disturbed by irregular flood pulses [32] and many studies have stressed that hydrological factors are more influential than physicochemical factors in the flood seasons [33,34]. However, long-term studies on zooplankton in FPLs are lacking, making it difficult to judge this view. Here, we followed the seasonal dynamics of zooplankton communities in the large subtropical FPL, Lake Poyang, during a 7-year period (2009–2016), characterized by large annual but rhythmic variations in water level. The purpose of this study was to determine the important mechanisms governing the seasonal dynamics of planktonic zooplankton. We hypothesized that the seasonal variation in water level/flow would be a key factor structuring zooplankton assemblages, as it affects the fish predation risk; the input of organic matter, which is a potential food source for zooplankton; and suspended matter concentration which affects the searching efficiency of visible hunting predators.

## 2. Materials and Methods

### 2.1. Study Area

**Figure 1:**
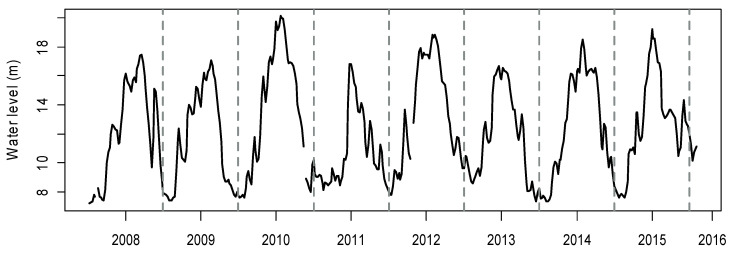
Weekly water level variation of Lake Poyang from 2008 to 2016.

Lake Poyang (115°49′–116°46′ E, 28°24′–29°46′ N) is China’s largest freshwater lake and is located in a subtropical wet climate zone characterized by an annual mean precipitation of 1680 mm and an annual mean temperature of 17.5 °C [35,36]. The lake receives water from five inflows: Ganjiang, Fuhe, Xinjiang, Raohe, and Xiushui, and lake water discharges into the Yangtze River. Among the inflows, the Ganjiang River contributes almost 55% of the total discharge into Lake Poyang [37]. The water residence time in the lake is mainly balanced by the inflow from the basin, the outflow, and water evaporation [38]. Only occasionally, there is a backflow from the Yangtze River to the lake, which transports suspended matter up to 20 km southward into the lake [39]. The annual water level variation can exceed 10 m within a year (Figure 1). The intra-year variations of water level were similar during the sampling years, being lowest in winter, increasing irregularly in spring, peaking in summer and decreasing abruptly in autumn (Figure 1). The inter-annual variation of the mean water level in the study years 2009–2015, was, respectively, 11.6 m, 13.6 m, 10.8 m, 13.6 m, 11.7 m, 12.6 m, and 12.8 m.

### 2.2. Sampling

**Figure 2:**
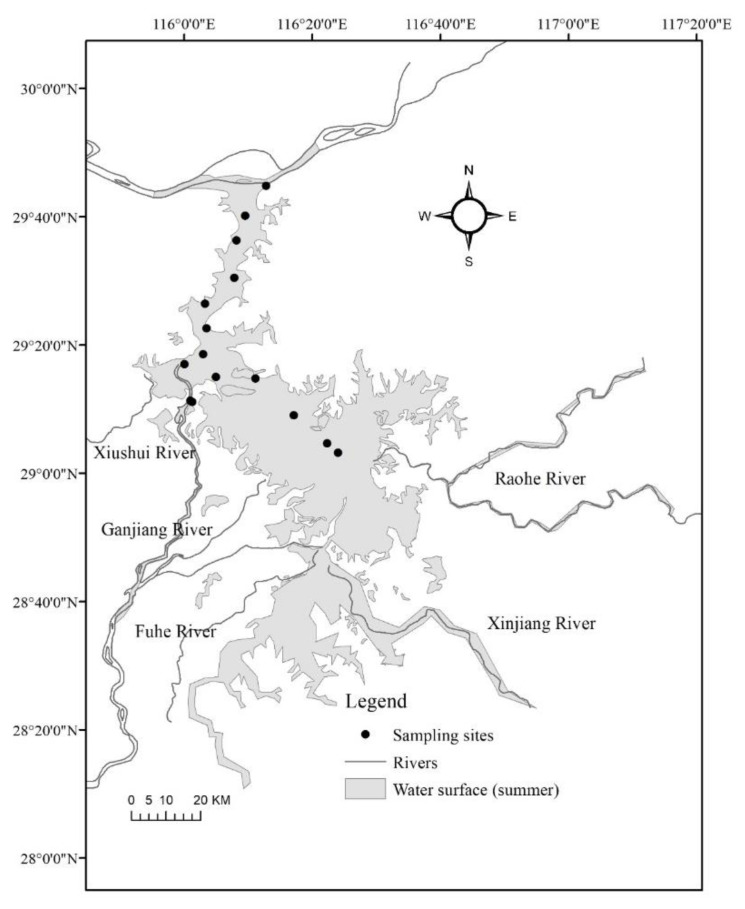
Sampling sites in Lake Poyang, China (trimonthly from winter 2009 to winter 2016). All sites were located in main channels that are inundated all year round.

Samples were collected quarterly from winter 2009 to winter 2016 at 15 stations along a south north gradient in the center of Lake Poyang (Figure 2). All samples were taken at the same site on the similar day in January, April, July, and October to represent winter, spring, summer, and autumn, respectively. All of the stations are located in deep channels that are flooded all year, while other portions of the lake that are only flooded in the summer are not studied. The mean flow velocity of the sites was >0.2 m/s, and water transparency was low (0.5 m), implying that vertical migration of zooplankton likely is insignificant. This was confirmed by Liu et al. [40], who found no significant differences in zooplankton biomass, size structure, and community composition between the different layers of the water column in the lake. We therefore only collected subsurface water at 0–1 m (to avoid anchoring the boat in the strong current) using a 5 liter (L) hydrophore. For crustacean zooplankton analysis, 10 L water was passed through a 64 μm mesh plankton net and preserved with 4% formalin.

Water temperature was measured using a Hydrolab DataSonda 5 Multiprobe (Hach Company, Loveland, CO, USA) in situ. Water transparency (Secchi depth) was measured using a Secchi disk. Suspended solids (SS), chlorophyll-a (Chl-a), chemical oxygen demand (COD), total nitrogen (TN), and total phosphorus (TP) were analyzed using standard methods according to APHA [41]. Water level data were extracted from the website of the Water Resources Department of Jiangxi Province (http://www.jxsl.gov.cn/slxxhw/jhsq/index.html, 1 July 2017) by Lake Poyang Laboratory for Wetland Ecosystem Research. Data on annual average air temperature and precipitation in the Lake Poyang basin were extracted from the yearbook of Jiangxi Province at the website of the Statistic Bureau of Jiangxi (http://www.jxstj.gov.cn/Column.shtml?p5=423, 1 July 2017).

### 2.3. Statistical Analysis

**Figure A1:**
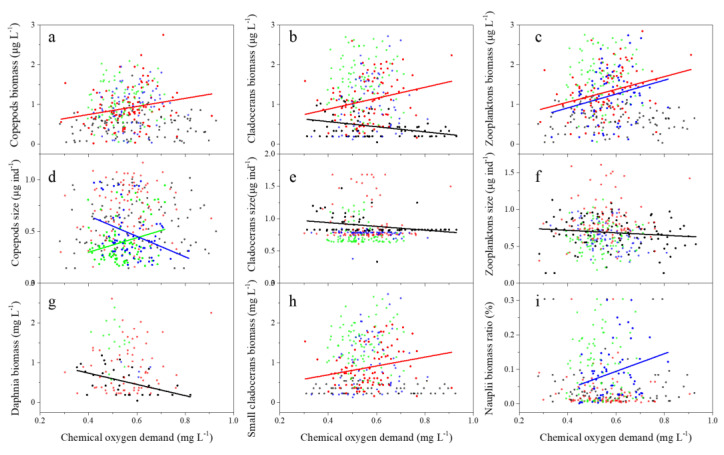
Relationship between total biomass and body size of various zooplankton species ((**a**) copepod biomass; (**b**) cladoceran biomass; (**c**) total zooplankton biomass; (**d**) copepod body size; (**e**) cladoceran body size; (**f**) zooplankton body size; (**g**) *Daphnia* biomss; (**h**) small cladoceran biomass; (**i**) napliui biomass ratio) versus chemical oxygen demand concentrations. Regression lines are shown only for significant relationships (*p* < 0.05). All data were log10(x + 1) transformed. Black = winter, red = spring, green = summer, blue = autumn.

We calculated zooplankton biomass according to body lengths and biomass regression equations applied by Liu [40]. The zooplankton mean body size was measured as the total biomass divided by a count of the individuals in each taxon. A total of 359 data points (100 data points for the winter and spring, 89 data points for the summer, and 70 data points for the autumn) were used in our analyses. Scatter plots and linear regression analysis were used to elucidate the effects of COD on zooplankton biomass and body size (Figure A1). The linear regression was separately performed for each season. Data were log10(x + 1) transformed when performing linear regression analysis. Partial least squares path modelling (PLS-PM) was applied using the raw data with the “plspm()” function in the package “plspm” to identify direct and indirect effects of water temperature and water level on zooplankton [42] and to analyze the strength of interactions [43].

All analyses were conducted with R3.4.1 (R Core Team, Hong Kong, 2017) Studio software packages.

## 3. Results

### 3.1. Inter- and Intra-Year Variations of Environmental Variables

**Figure 3:**
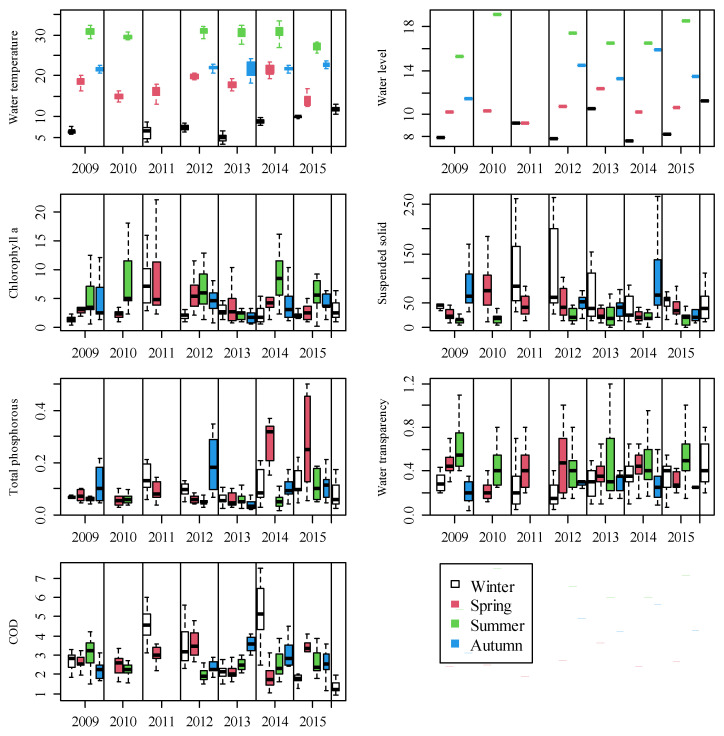
Boxplots (median, 10, 25, 75, and 90 percentiles) showing the seasonal dynamics in water temperature (°C), water level (m), chlorophyll a (μg L^−1^), suspended solids (mg L^−1^), total phosphorus (μg L^−1^), water transparency (m), and COD (chemical oxygen demand, mg L^−1^) during the study periods.

Large seasonal variations of physiochemical environmental variables were found (Figure 3). The seasonal fluctuations in water temperature, water level, and Chl-a concentrations were all similar, with summer being the highest, followed by autumn and spring, and winter being the lowest. Generally, suspended solids (SS) concentrations peaked in winter and exhibited minimum concentrations in summer. Water transparency was highest in summer and lowest in winter. TP concentrations were lowest in summer, but high concentrations also appeared in winter, spring and autumn. The time of peak and low values of COD varied between the years.

There was a significant increasing trend with time for water temperature (*p* = 0.04, R^2^ = 0.52) and water transparency (*p* = 0.05, R^2^ = 0.46) in winter, while there were no significant trends for any of the environmental variables in spring or autumn.

### 3.2. Zooplankton Biomass and Body Size

**Figure 4:**
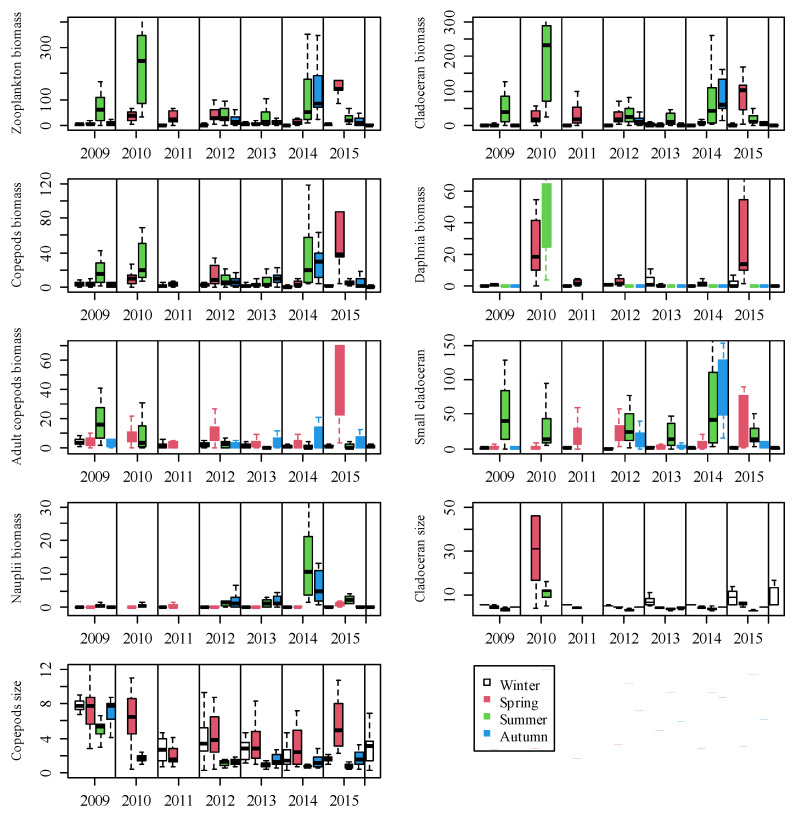
Boxplots (median, 10, 25, 75, and 90 percentiles) showing the seasonal dynamics of zooplankton, cladocerans, copepods, *Daphnia*, adult copepods, small-bodied cladocerans and nauplii biomass (μg DW L^−1^) and mean cladoceran and copepod body size (μg ind^−1^) during the study periods. Note: Some values appear outside the figure frame to clearly display the general trends.

**Figure 5:**
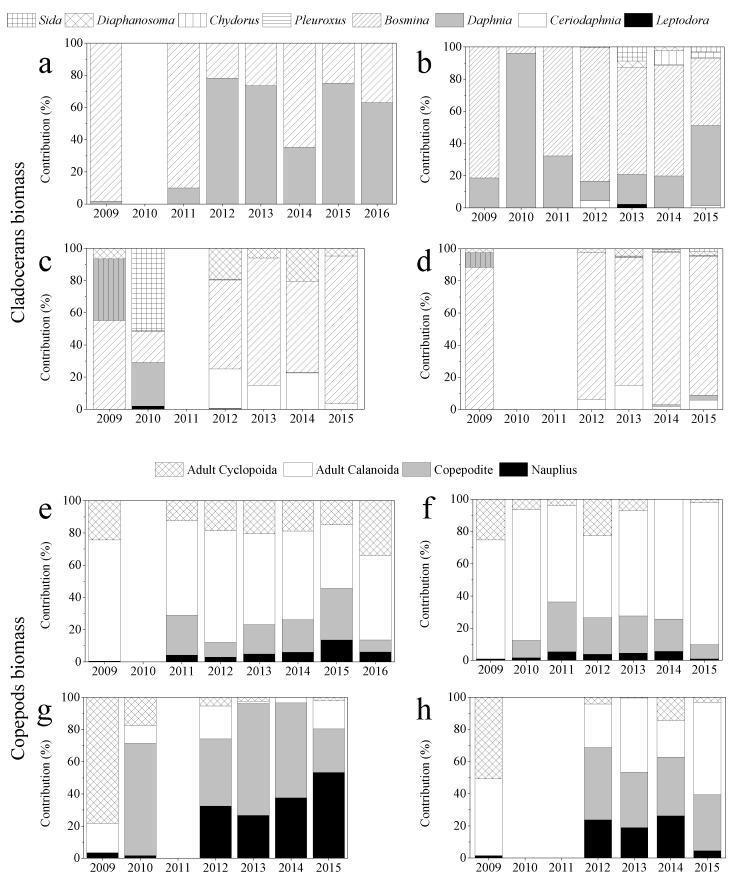
Cladoceran and copepod biomass dynamics in (**a**/**e**) winter, the low water level season, (**b**/**f**) spring, the rising water level season, (**c**/**g**) summer, the high-water level season and (**d**/**h**) autumn, the decreasing water level season.

Zooplankton biomass was lowest in winter and most often highest in summer (Figure 4). Cladoceran biomass peaked in spring 2015; summer 2009, 2012 and 2013; and autumn 2014; Copepod biomass peaked in spring 2012 and 2015, summer 2009, and autumn 2013 and 2014. *Daphnia* biomass usually reached maximum values in spring or sometimes in winter but was low in summer and autumn. The biomass of adult copepods peaked in spring or autumn and was always low in winter. The biomasses of *Daphnia* and adult copepods were both relatively high in spring and summer 2010 and 2015 when the water temperature was particularly low (see also Figure 3 and Figure 5). The biomass of small-bodied cladocerans was lowest in winter and most often highest in summer or occasionally in spring (2015) or autumn (2014). Nauplii biomass was lowest in winter and highest in summer. Both small-bodied cladoceran biomass and nauplii biomass were relatively high in summer and autumn 2014. Generally, mean cladoceran body size was highest in winter and lowest in summer, while the highest mean body size of copepods was found in spring and the lowest in summer.

### 3.3. Zooplankton Community Structure

Large-bodied *Daphnia* constituted a higher proportion of the cladoceran biomass in winter and spring than in the other seasons. *Daphnia* represented >50% of the cladoceran biomass in winter 2010, 2012, 2013, 2015, and 2016, while in spring *Daphnia* constituted a large part of the cladoceran biomass only in 2010 and 2015 when the water temperature was particularly low (Figure 5). Furthermore, *Daphnia* and other large-bodied cladocerans almost disappeared from the lake in summer, except for 2010. In contrast, small-bodied cladocerans were more abundant in summer and autumn (Figure 5). In autumn, Bosmina made up >80% of cladoceran biomass in most years. Similarly, adult copepods contributed more to copepod biomass in winter and spring than in summer and autumn. In contrast, juvenile copepods constituted a greater proportion of the copepod biomass in summer and autumn.

### 3.4. Direct and Indirect Effects of Water Temperature and Water Level

**Figure 6:**
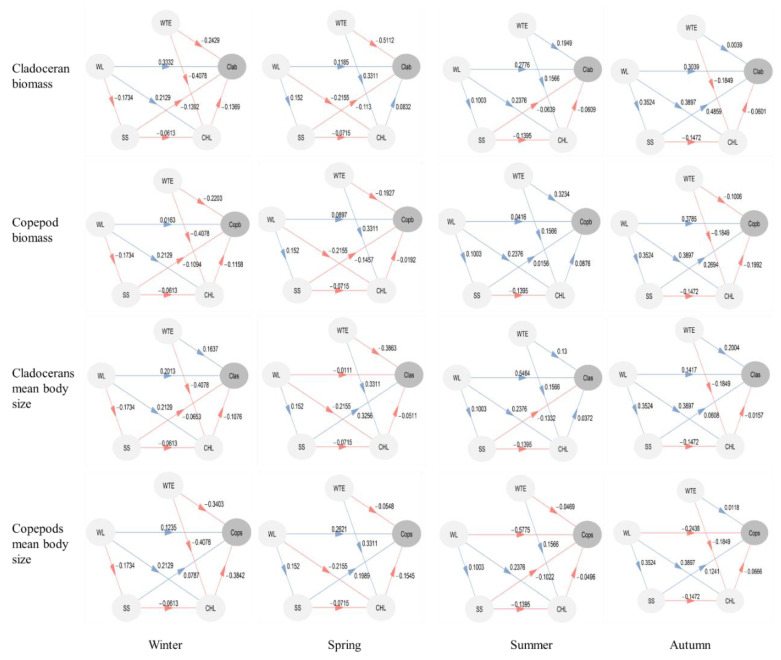
Results of partial least squares path modeling (PLS-PM) analysis of the direct and indirect effects of water temperature and water level on crustacean zooplankton biomass and body size in different seasons. (Clab = cladoceran biomass, Copb = copepod biomass, Clas = cladoceran mean body size, Cops = copepod mean body size, WTE = water temperature, WL = mean water level five weeks before and during sampling, respectively, SS = suspended solids concentration, CHL = chlorophyll a concentration).

Some of the environmental variables in the lake were correlated with each other in the different seasons; for example, Chl-a concentrations had a significant negative relationship with water temperature in winter (*p* < 0.01) but a positive relationship in spring (*p* = 0.01). Therefore, we analyzed the direct and indirect effects of temperature and water level on the biomass and mean body size of zooplankton using PLS-PM (Figure 6). According to this analysis, water temperature had negative direct effects on cladoceran biomass, copepod biomass, and copepod mean body size during winter but positive direct effects on cladoceran mean body size; Water level being the dominant factor positively affecting cladoceran biomass and mean body size. Water temperature affected Chl-a concentrations more strongly than water level, these two being the dominant factors directly controlling copepod mean body size.

During spring, the season in which the water level increased, water temperature was the dominant factor negatively affecting cladoceran biomass, cladoceran mean body size and copepod biomass. Direct effects of water level on crustacean zooplankton biomass and body size were weak. However, water level had an indirect effect on the zooplankton through SS, which had negative direct effects on cladoceran and copepod biomass but positive effects on their mean body size. Copepod mean body size was directly affected by water level, SS and Chl-a in spring, and water level and water temperature indirectly affected copepod body size through both Chl-a and SS.

During summer, when the lake had the largest water surface area, water level was the dominant factor positively affecting cladoceran biomass and body size but negatively affecting copepod mean body size, while water temperature was the dominant factor positively influencing copepod biomass.

During autumn, when the water level was rapidly decreasing, water level was the dominant factor positively affecting copepod biomass but negatively influencing copepod mean body size, SS was the dominant factor positively affecting cladoceran biomass, and water temperature was the dominant factor having a positive effect on cladoceran mean body size. Furthermore, water temperature had a negative effect on copepod biomass and indirectly affected it via Chl-a. Water level had positive impacts on cladoceran biomass and indirectly affected crustacean biomass and mean body size through SS and Chl-a.

## 4. Discussion

Our results indicated that zooplankton biomass and body size during all seasons were directly and indirectly affected by hydrological regime factors, such as water level and suspended solids. Compared with subtropical lakes in the classical PEG model [5,28], our study lake was characterized by a shorter retention time. Here we discuss the findings season by season.

In winter, we found a re-set of the phytoplankton Chl-a to low concentrations as in temperate lakes but opposite to tropical lakes (e.g., two South African lakes in the original PEG database and six Mediterranean lakes in one of the modified PEG models) [44]. We did not find a positive relationship between zooplankton biomass and Chl-a as otherwise predicted by the PEG model [5,28], indicating that phytoplankton was not the main driver of zooplankton in winter. The decomposed hygrophytes during this season may have offered additional food sources for zooplankton. Allochthony (allochthonous sources from both dissolved and particulate organic matter) of freshwater crustacean zooplankton has been demonstrated in bottle experiments [45], in situ mesocosm studies [46], and whole-lake investigations [47,48].

Moreover, we found a significant increasing trend for cladoceran mean body size in winter during the study period, coinciding with a significant increasing water temperature, likely illustrating the crucial impact that water temperature has on cladocerans in the subtropical winter [29,49]. The trend, however, was somewhat different for the biomass and mean body size of all crustaceans pooled due to a reduction in biomass of copepods. Perhaps the increasing cladoceran biomass may have resulted in a competitive reduction in copepods, as the food was dominated by allochthonous sources. For example, Berggren and Ziegler [50] revealed allochthony of Cladocera, Calanoida and Cyclopoida using a multi-isotope (δ^2^H + δ^13^C) approach across 18 lakes in Quebec and found that Cladocera had the highest degree of allochthony (0.31 compared with 0.18 for Cyclopoida and 0.16 for Calanoida).

Water level variation in spring appeared to have a smaller impact on the zooplankton biomass pattern than water temperature. Zooplankton biomass and mean body size were predominantly linked to water temperature, which is congruent with the results of other studies testing the PEG model [29]. High concentrations of SS, caused by the fast flow, may have played a role as we found a negative relationship between zooplankton biomass and SS, while the COD concentration (potential food resource, Figure A1) showed a significant positive relationship with the biomass of crustacean. Possibly, this indicates that allochthonous sources of decomposed hygrophytes and organic sediment (when flooded in spring) may be an additional food source in spring for part of the crustacean zooplankton taxa [51,52], thereby modulating the direct link between phytoplankton (Chl-a) and zooplankton (as we found no relationships between the latter two in spring). The mean dissolved organic carbon in April 2014, for example, reached up to 4.35 mg/L (unpublished data), while corresponding mean phytoplankton carbon was about 1.5 mg/L (3.28 mg/L total phytoplankton biomass).

Crustacean biomass always peaked in summer when the water level and Chl-a concentrations were at their highest. The peaks of water clarity may encourage the peak of chlorophyll-a, and the two together facilitate the peaks of crustacean biomass (physically and biologically).The PEG model, on the other hand, predicts a lower crustacean biomass in summer, which is explained by higher fish predation when juvenile fish occur in high densities. We also found indications of increased fish predation as the crustacean zooplankton had the lowest mean body size in summer, but the pattern was less strong than that outlined in the PEG model, likely because high water level to some extent counteracted the summer increase in predation [40]. Indeed, our PLS-PM analysis demonstrated that biomass of the cladoceran positively correlated with the water level. An additional factor contributing to a higher crustacean biomass in summer may be passive advective transport of zooplankton from off-channel lentic limnetic habitats to the main channel, as earlier shown for phytoplankton in Lake Poyang [53]. Advective transport has been found also in other studies of FPL [11,54,55].

While the predation pressure by planktivorous fish is seasonally high in late spring and early summer in the temperate zone, our results indicate a high predation pressure on large-bodied zooplankton also in autumn in subtropical lakes, which may reflect the strongly decreasing water level during this period, implying an increasing fish biomass per m^3^ [56]. A high predation pressure is evidenced by the almost absence of large-bodied *Daphnia* and, moreover, the mean body size of copepods and cladoceran was second lowest in autumn in nearly all years. In addition, *Bosmina,* which is less sensitive to planktivorous fish predation than *Daphnia* [57], constituted more than 80% of the cladoceran biomass during autumn. Furthermore, crustacean zooplankton biomass and mean body size were significantly positively related to suspended solid concentrations (mainly caused by turbulence in autumn), which is in accordance with a lower predation pressure by visibility-dependent planktivores under turbid conditions [58,59,60].

## 5. Conclusions

Our results demonstrate that hydrological factors may be important drivers of the seasonality of zooplankton in FPLs (Table 1). This was most evident in (1) spring when the water level increases and hydrophytes areas are inundated, making allochthonous sources available to the zooplankton, and in (2) autumn when the water level decreases and the water volume shrinks, expectedly increasing the predation pressure on zooplankton. Results from other hydrology-influenced lake systems are, however, needed to be able to draw firm conclusions that can extend the PEG model to such lake types.

## Figures and Tables

**Table 1:** Similarities and differences in the seasonal variation pattern of planktonic crustaceans between common lakes described in the classical PEG model and a floodplain lake—Lake Poyang.

	As in PEG Model	Differences from PEG Model and Additions
Winter	High food limitation	Negative effects of temperature and chlorophyll-a on biomass Positive relationships between water level with cladoceran and *Daphnia* biomass
Spring	Water temperature the dominant factor High grazing on phytoplankton	Positive relationship with COD instead of chlorophyll-a concentration
Summer	High predation pressure	Biomass peaks in summer Potential passive advection migration (similar to phytoplankton) (Liu et al., 2015)
Autumn	Not clear	High predation pressure

## Data Availability

Data sharing is not applicable to this article.
